# Clinical feature and genetic mutation of KBG syndrome diagnosed in neonatal period: A case report

**DOI:** 10.1097/MD.0000000000035449

**Published:** 2023-10-06

**Authors:** HaoZheng Zhang, Xuening Guo, Chun Yang, Kaihui Zhang, Dong Wang, Juan Wang, Yi Liu, Lili Kang, Qinghua Liu, Xiaoying Li

**Affiliations:** a Pediatric Research Institute, Children’s Hospital Affiliated to Shandong University, Jinan, China; b Neonatal Intensive Care Unit, Children’s Hospital Affiliated to Shandong University, Jinan, China; c Department of Clinical Laboratory, Children’s Hospital Affiliated to Shandong University, Jinan, China; d Department of Ultrasound, Children’s Hospital Affiliated to Shandong University, Jinan, China.

**Keywords:** ANKRD11 gene, case report, KBG syndrome, recurrent infection

## Abstract

**Rationale:**

KBG syndrome (KBGS, OMIM: 148050), a rare genetic disorder, is clinically characterized by megalodontia, short stature, skeletal abnormalities, and nervous system manifestations. In the study, we explore the clinical and genetic characteristics of one neonate suffering KBGS caused by ANKRD11 gene mutation.

**Patient concerns:**

The proband, a female, was born prematurely at 31 + 2 weeks. There were repeated infections and abdominal distension in the first month after birth, and the platelets could not rise to normal. Head ultrasound showed intracranial brain injury and intracranial hemorrhage.

**Diagnoses:**

Sequencing revealed that there was a heterozygous mutation in exon 9 of the ANKRD11 gene (NM_013275.5) for the child, c.1896_1897delTA (p.H632Qfs*30), which was a de novo mutation and has not been reported. Combining clinical features and genetic results, the proband was diagnosed as KBGS.

**Interventions and outcomes:**

The brain sonography on day 4 after birth showed brain injury and intracranial hemorrhage. Therefore, 140 mg of bovine lung surfactant was administered through endotracheal intubation in addition to ventilator-assisted ventilation. Antibiotic treatment was also given till the inflammatory indicators of the infant returned to normal levels. The following-up of 1-year-6-month showed that the language, motion and height of development is slight falling behind the children of the same age.

**Lessons:**

This is the first case of KBGS was diagnosed in the neonatal period, which provides a reference for the child to receive timely and correct treatment.

## 1. Introduction

KBG syndrome (KBGS, OMIM: 148050), a rare genetic disorder, is clinically characterized by megalodontia, short stature, skeletal abnormalities, and nervous system manifestations representative by generalized developmental delays, seizures, and intellectual disabilities in accompany with developmental delays, learning disabilities, or significant behavioral abnormalities such as autism.^[1,2]^ According to the literature, this syndrome was first reported by Hermann et al as early as 1975.^[3]^ With the increasing application of second-generation sequencing technology in the diagnosis of genetic diseases, the pathogenic gene of this syndrome, ANKRD11, was eventually identified by Sirmaci et al in 2011.^[4]^ ANKRD11 gene, as an autosomal dominant gene, is located in chromosome 16q24.3 and contains 11 exons. Its encoding protein, consisting of 2663 amino acids, is a protein containing ankyrin repeat cofactor, which can inhibit ligand-dependent transcriptional activity by binding to p160 nuclear receptor coactivator.

Based on the diagnostic criteria of KBGS revised in 2016, a clinical diagnosis of KBGS can be confirmed for cases with 2 major indicators or one major indicator combined with 2 secondary indicators. The major indicators include large maxillary central incisor, short stature, recurrent otitis media with or without hearing loss, and KBGS identified among first-degree relatives; The secondary indicators include short fingers or associated hand abnormalities, epilepsy, cryptorchidism, feeding difficulties, abnormal palatal arch, autism, as well as large fontanel or delayed closure.^[5]^

So far, over 200 cases of KBGS have been reported globally, including less than 10 cases recorded in China,^[1–6]^ none of which, however, was diagnosed in the neonatal period. In the present study, we analyzed the clinical manifestations and gene reports of a neonatal patient with KBGS, in an attempt to improve the understanding on the characteristics associated with the clinical phenotype and gene mutation of this disease.

## 2. Data and methods

### 2.1. Clinical data

In July 2021, a 1-month-old female newborn was referred to the Neonatal Intensive Care Unit, Department of Neonatology, Affiliated Children’s Hospital of Shandong University, due to “premature delivery at 31 + 2 weeks, recurrent infection for 1 month, and abdominal distension for half a day.” Detailed physical examination of clinical signs and comprehensive laboratory tests were performed, followed by cranial and abdominal ultrasonography, as well as genetic tests and analysis. This study was approved by the Ethics Committee of Qilu Children’s Hospital of Shandong University (approval number: QLET-IRB/P-2021071).

### 2.2. Methodology

The proband was subsequently diagnosed as KBGS, a rare genetic disease. Among all the research methods, genetic tests were highlighted in this study, with the procedures delineated as follows.

1. With the consent obtained from the parents of the newborn, 1 and 5 mL of peripheral blood were collected respectively from the patient and her parents. After EDTA anticoagulation treatment, the blood samples were used for further molecular detection. A drop of blood was taken to prepare dry blood spots for amino acid and fatty acid determination, followed by urine collection for organic acid test.
2. In the test for identifying genetic and metabolic diseases, gas chromatograph-mass spectrograph (Shimadzu GCMS-QP2010 Ultra) and relevant analytical software (Shimadzu newborn screening-2) were employed for pre-processing, derivatization, and outcome comparison of urinary organic acids. By contrast, these procedures for blood amino acids and fatty acids testing were performed by liquid chromatograph-mass spectrograph (AB SCIEX API 3200MD) equipped with supporting analysis software.
3. Whole genome detection was conducted by chromosome microarray (Affymetrix CytoScan HD, Santa Clara, CA), which was applied in the procedures of amplification, purification, hybridization, washing and scanning of the extracted genomic DNA. Besides, Chromosome Analysis Suite was employed for analysis and annotation of the single nucleotide polymorphism, and databases GRCh38/hg19, OMIM, DECIPHER, as well as ISCA were used for evaluation on genotypic-phenotype correlation.
4. Whole exon sequencing for disease-related genes was performed in Beijing Fujun Genetics Co., Ltd. The genomic DNA extracted from the samples, after going through the procedures of fragmentation, gene-splicing, as well as amplification and purification, was employed to establish DNA libraries using hybridization capture technique. Afterwards, a high-throughput sequencing platform novaseq6000 was used to detect the exon region and lateral intron region of the 20,099 genes in the human whole exome (20 bp). By comparing the sequencing data with the reference sequences of human genome hg19, the coverage of the target region and the quality of the sequencing procedure were evaluated (the parameters of the patient are shown in the table below). Evaluation on the pathogenicity of the variants was carried out with reference to the practice guideline (2015) from the American College of Medical Genetics and Genomics. Sanger sequencing was performed for validation of generation sequencing procedure.

## 3. Results

### 3.1. Clinical feature

The study reported a 1-month-old female proband with Han nationality whose non-consanguineous parents were healthy with no family history of genetic disorders or contagious diseases. She was the first birth from the 23-year-old mother, who was diagnosed as gestational hypertension at 5 months’ gestation. During pregnancy, 2 tests for Down’s syndrome suggested high potential risk, in contrast to the results of noninvasive prenatal testing that indicated no abnormalities in the fetus. At 31 + 2 weeks’ gestation, an emergency cesarean section was performed in People’s Hospital of Yuncheng County due to severe preeclampsia, severe placental abruption with a separation area of 4/5, and slow fetal heart rate. Neonatal physical examination recorded a birth weight of 1.18 kg and a heart rate of 70 bpm, together with birth asphyxia, bluish discoloration on the skin, flaccid limbs, and unresponsiveness. Medical intervention was carried out immediately, including warmth keeping, airway clearing, and 30 seconds of positive pressure ventilation using resuscitation device. The subsequent examination revealed improved muscular tone, with the heart rate recorded as >100 bpm and 1-minute Apgar score rated as 5. But crying was still not observed in this newborn. No abnormalities were detected in amniotic fluid and umbilical cord. Blood tests indicated significantly increased procalcitonin (100 ng/mL) as well as relatively low platelet count (124 × 10^9^/L) accompanied by coagulation dysfunction as demonstrated by difficulty in stopping the bleeding on the puncture sites. The brain sonography on day 4 after birth showed brain injury and intracranial hemorrhage. Therefore, 140 mg of bovine lung surfactant was administered through endotracheal intubation in addition to ventilator-assisted ventilation. Antibiotic treatment was also given till the inflammatory indicators of the infant returned to normal levels. During the hospitalization, recurrent fever was noted 4 times during 1 month, accompanied by elevated levels of white blood cells, C-reactive protein and procalcitonin. Besides, 2 apnea episodes were also observed within 27 days.

*Physical examination*: T 37.0°C, P 160 bpm, R 60 breaths/min, BP 75/42 mm Hg, WT 1.4 kg. The immature infant was malnourished with poor response and tachypnea. The inspection revealed less ruddy skin with an uneven texture, and scattered petechiae on the limbs caused by venipuncture. The anterior fontanel, which remained unclosed, measured about 2 cm × 2 cm. Perioral cyanosis and the congestion in pharyngeal mucosa were also noted in the examination. Retraction sign of 3 fossae was negative. Pulmonary auscultation suggested coarse breathing sounds in bilateral lungs without rhonchi or moist rales. A stable heart rate of 160 bpm along with strong cardiac sounds was registered, with no murmurs heard in any of the auscultatory valve areas. Abdominal palpation indicated abdominal distension and mild muscular hypertonicity. The abdominal circumference was measured as 27 cm. Abdominal compression did not elicit crying or painful expressions. No mass was spotted in abdominal palpation, by which the liver and spleen were also not touched. The bowel sounds were 3 times/min. Examination of the fingers suggested normal capillary filling time and nail-bed pallor but with no cyanosis. No abnormality was found in muscle tone test of the extremities. The clinical diagnosis was eventually confirmed as neonatal sepsis, anemia, and thrombocytopenia.

**Figure 1.**
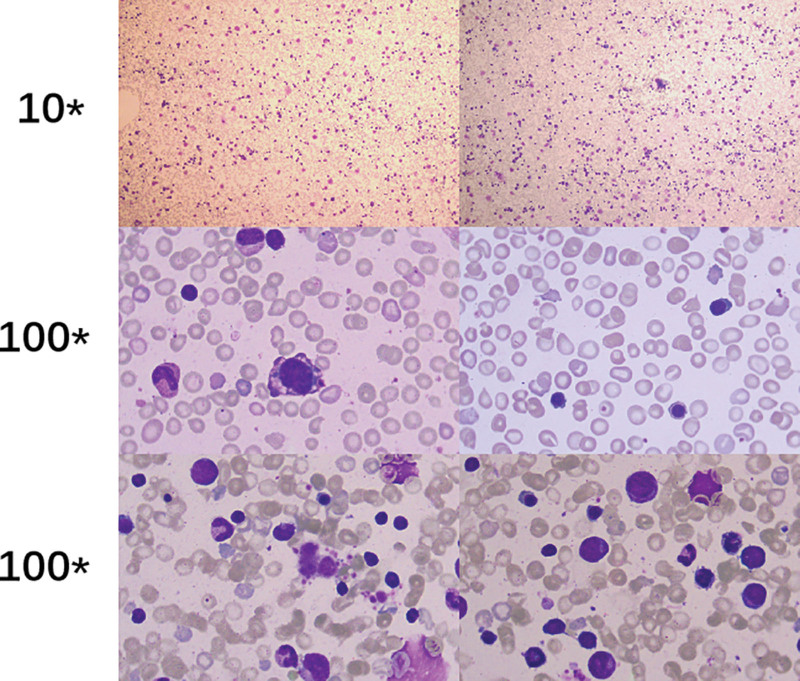
Bone marrow smear test suggests significantly active bone marrow hyperplasia, with lymphocytes accounting for 48%, juvenile lymphocytes for 2.5%, granulocytes for 30%, and erythrocytes for 21%. In the myelogramme, all stages of granulocytes are coexisted, with a higher ratio of neutrophilic myelocytes observed, while polychromatic and orthochromatic normoblasts were predominant in erythrocytic series. 10* and 100* represent the magnifications of the objective lens.

*Laboratory examination*: Repeated blood routine tests all showed increased leukocyte levels beyond the upper limit of normal range, with the highest recorded as 28.67 × 10^9^/L, which was mainly attributed to elevated counts of lymphocytes. The results also revealed lifted C-reactive protein levels as high as 46.50 mg/L and lowered platelet counts down to 45 × 10^9^/L. The infant suffered sustained anemia, as demonstrated by declined erythrocyte counts as low as 2.79 × 10^12^/L and the decrease in hemoglobin with a minimum value of 75 g/L. Bone marrow smears suggested significantly active bone marrow hyperplasia, with lymphocytes accounting for 48%, juvenile lymphocytes for 2.5%, granulocytes for 30%, and erythrocytes for 21%. In the myelogramme, all stages of granulocytes were coexisted, with a higher ratio of neutrophilic myelocytes observed, while polychromatic and orthochromatic normoblasts were predominant in erythrocytic series (Fig. 1). Besides, no anomaly was detected pertaining to the thyroid function, as well as the levels of immunoglobulin, antinuclear antibody, 25-hydroxyvitamin D and urinary calcium/creatinine ratio.

**Figure 2.**
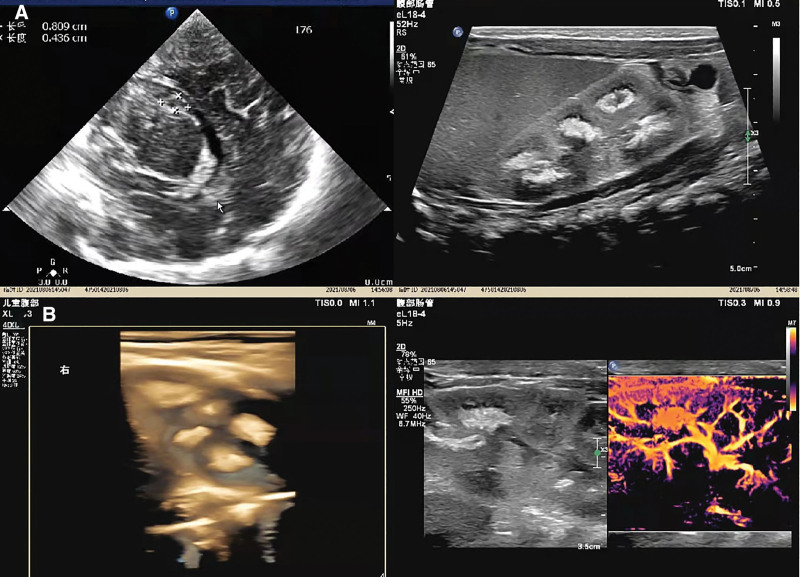
Imagological and other auxiliary examinations. (A) Ultrasonography indicates the absorption period of bilateral subependymal hemorrhage. The sagittal view through the fontanel reveals a hyperechoic area measuring about 0.8 × 0.4 cm in the left subependymal lesion, with a sac-like anechoic zone inside. The bilateral bodies of lateral ventricles are both about 0.2 cm in width. The coronal view indicates sharp anterior horns of the lateral ventricles. The width of the third ventricle measures about 0.3 cm. The thalamus, caudate nucleus and lenticular nucleus are clearly defined. No abnormality was spotted in the echoes of both cerebral hemispheres. (B) Ultrasonography suggests bilateral medullary sponge kidneys. The left kidney measures about 5.0 × 2.2 cm, and the right kidney about 4.2 × 2.2 cm. Homogeneous echoes are detected from the renal cortex. However, diffuse hyperechoic mass was spotted around the collecting system in bilateral medulla kidneys, with no evident posterior shadow observed. There is no noticeable separation of bilateral collecting systems or dilatation of bilateral ureters. CDFI indicates normal blood perfusion in both kidneys. CDFI = color doppler flow imaging.

Imagological and other auxiliary examinations: Imaging examinations such as magnetic resonance imaging and computerized tomography are usually restricted in neonatal application due to their limitations, including the use of sedatives, high cost, and radiation. By contrast, color ultrasonography can be used to fill the gaps in the diagnosis of neonatal diseases. In this case, cranial and thoracoabdominal ultrasonography was employed for imaging examinations. Cranial ultrasonography showed grade I intracranial hemorrhage (Fig. 2A). Renal ultrasonography suggested separation of the left renal collecting system (Fig. 2B). In addition, bone marrow smear indicated: thrombocytopenia; infectious anemia combined with nutritional anemia; and hemolytic anemia to be further confirmed (Fig. 2C).

The following-up of 1-year-6-month showed that the language, motion and height of development is slight falling behind the children of the same age.

### 3.2. Genetic detection and pathogenicity analysis of the variants

**Figure 3.**
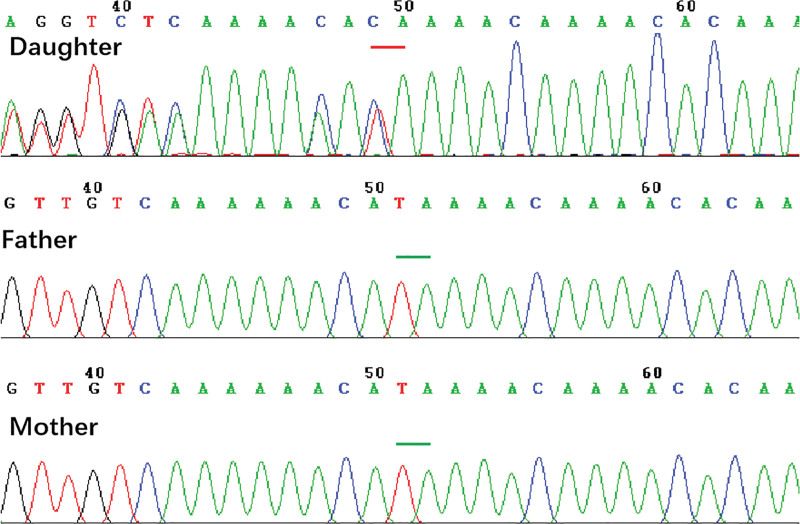
The mutation was verified by Sanger sequencing. There is a mutation, c.1896_1897delTA, in exon 7 of ANKRD11 gene on chromosome 16 which is a de novo and novel mutation.

The tests for genetic metabolic diseases, including analysis of urine organic acids, blood amino acids and fatty acids, revealed no abnormality, nor did noninvasive prenatal testing or whole-genome microarray test. Whole exon sequencing indicated a mutation in exon 7 of the ANKRD11 gene on chromosome 16: c.1896_1897delTA (p.H632Qfs*30), which was verified as a novel mutation by Sanger sequencing due to its absence from both of the parents. Considering this mutation has not been reported in the literature, it was eventually identified as a de novo variant (Fig. 3). This variant, as a frameshift mutation, can cause alterations in the open reading frames of the gene, thus resulting in abnormal synthesis of the polypeptide chains of proteins. According to the American College of Medical Genetics and Genomics criteria, the c.1896_1897delTA mutation was defined as a pathogenic mutation (PVS1 + PS2 + PM2). The majority of the variations in ANKRD11 gene included in the literature and the database were frameshift mutations, and c.1896_1897delTA identified in this study was also frameshift mutations, which is consistent with the previous reports.

## 4. Discussion

Ankrd11, a potential chromatin regulator, is associated with neurodevelopment and autism spectrum disorder.^[7,8]^ Knockdown of Ankrd11 expression in mice can slow down the proliferation and production of embryonic cortical neuron precursors, and lead to abnormal localization of neurons.^[9,10]^ Studies have shown that Ankrd11 is in connection with chromatin acetylation and involves in colocalization with HDAC3. It is known that Ankrd11 knockdown results in the decreased proliferation of neuronal precursors, however, the phenotypes caused by Ankrd11 knockdown could be rescued by inhibiting histone acetyltransferase or enhancing HDAC3 expression.^[9]^ Therefore, Ankrd11, being recognized as a key chromatin regulator controlling histone acetylation and gene expression during neurodevelopment, may offer a possible interpretation on its association with cognitive dysfunction and autism spectrum disorder. Quantitative analysis of craniofacial feature of the patients with KBGS using 3D imaging technique showed that the overall size of the mid-face and lower face reduced in these cases,^[11–13]^ which is similar to the phenotype of Robinow syndrome caused by core gene mutation in the Wnt/PCP signaling pathway. The dynamic changes in histone acetylation during ossification are associated with the key osteogenic factors such as Runx2, Sp7, and Alp. Given that Runx2 can directly interact with HDAC3, Ankrd11 and Runx2 may be involved in a common gene regulatory network, jointly playing a role in epigenetic regulation during craniofacial bone development.^[11,14]^

By February 2021, 232 cases of Ankrd11 gene mutation had been included in the professional version of HGMD database, among which 161 gene mutations lead to KBGS, 6 gene mutations resulted in Cornelia de Lange syndrome,^[15–17]^ 11 mutations caused autism-related symptoms, and another handful of variations were associated with abnormalities in the development of the nervous system or organs. So far, previous studies have reported 2 cases of KBGS whose mutations c.1893dup (P.IS632thter2) and c.1903_1907del (p.Lys635fs Ter26) are similar to that found in this study in terms of locations and properties. Moreover, c.1903_1907del, as a common hot spot mutation,^[9]^ was found in 6 patients with KBGS. Nevertheless, the KBGS caused by c.1893dup (p. His632Thr Ter2) and c.1903_1907del (p. Lys635fs Ter26) variations reported in the literature was found in adolescence and adulthood. By summarizing their clinical manifestations and comparing them with the findings of our case, the child was eventually confirmed as KBGS accompanied by severe sepsis in the neonatal period. As is known, KBGS is prone to be complicated by infectious diseases, so the patient’s intractable severe sepsis is speculated to be aggravated by the mutation of Ankrd11 gene.

### 4.1. Novel insights

It was not uncommon that KBG was diagnosed in childhood, while this case is the first KBGS diagnosed in the neonatal period. Typically, children with this disease will end up with a short stature in the future, whereas, growth hormone treatment may improve the symptoms, particularly, the use of growth hormone in the early stage can contribute to the improvement of the final height of patients with KBG. Also, children with KBGS are usually subject to concurrent infection, especially refractory severe neonatal sepsis collectively induced by some factors during pregnancy. In such cases, exclusion of underlying diseases becomes the priority of clinicians. Inherited chimerism should be taken into consideration, and prenatal diagnosis is also an effective way to prevent recurrence of the disease in the family.

## Acknowledgments

The authors are grateful to the patients and their parents for their contribution to this study.

## Author contributions

**Data curation:** HaoZheng Zhang, Lili Kang.

**Formal analysis:** Xuening Guo, Dong Wang.

**Funding acquisition:** HaoZheng Zhang, Kaihui Zhang, Xiaoying Li.

**Investigation:** HaoZheng Zhang, Kaihui Zhang.

**Methodology:** Chun Yang, Juan Wang.

**Project administration:** HaoZheng Zhang, Yi Liu.

**Resources:** Chun Yang, Lili Kang, Qinghua Liu.

**Writing – original draft:** HaoZheng Zhang, Xuening Guo.

**Writing – review & editing:** HaoZheng Zhang, Chun Yang, Xiaoying Li.
